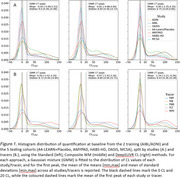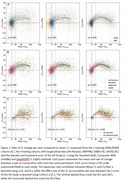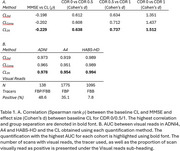# Using AI to improve cross‐sectional and longitudinal Amyloid PET quantification: A validation study across 7 large cohorts

**DOI:** 10.1002/alz70856_104230

**Published:** 2025-12-24

**Authors:** Pierrick Bourgeat, Jurgen Fripp, Ashley G Gillman, Timothy Cox, Azadeh Feizpour, Ying Xia, Georgios Zisis, Manu S. Goyal, Duygu Tosun, Pamela LaMontagne, Tammie L.S. Benzinger, Frederik Barkhof, Gill Farrar, Ariane Bollack, Reisa A. Sperling, John C. Morris, Michael S. W. Weiner, Victor L. Villemagne, Colin L Masters, Christopher C. Rowe, Vincent Dore, the ADNI, OASIS3, AMYPAD Consortium, Mayo Clinic Study of Aging and the AIBL research group

**Affiliations:** ^1^ CSIRO Health and Biosecurity, Australian E‐Health Research Centre, Brisbane, QLD, Australia; ^2^ CSIRO Health and Biosecurity, Australian E‐Health Research Centre, Townsville, QLD, Australia; ^3^ The Australian e‐Health Research Centre, Commonwealth Scientific and Industrial Research Organisation, Melbourne, VIC, Australia; ^4^ Department of Molecular Imaging & Therapy, Austin Health, Melbourne, VIC, Australia; ^5^ Florey Institute of Neuroscience and Mental Health, Parkville, VIC, Australia; ^6^ Mallinckrodt Institute of Radiology, Washington University School of Medicine, St. Louis, MO, USA; ^7^ University of California, San Francisco, San Francisco, CA, USA; ^8^ Washington University School of Medicine, Saint Louis, MO, USA; ^9^ Department of Radiology and Nuclear Medicine, Vrije Universiteit Amsterdam, Amsterdam University Medical Center, location VUmc, Amsterdam, Netherlands; ^10^ GE HealthCare, Amersham, United Kingdom; ^11^ Center for Alzheimer Research and Treatment, Brigham and Women's Hospital, Boston, MA, USA; ^12^ Knight Alzheimer Disease Research Center, Washington University School of Medicine, St. Louis, MO, USA; ^13^ VA Advanced Imaging Research Center, San Francisco Veterans Affairs Medical Center, San Francisco, CA, USA; ^14^ University of Pittsburgh School of Medicine, Pittsburgh, PA, USA; ^15^ The Florey Institute of Neuroscience and Mental Health, The University of Melbourne, Parkville, Melbourne, VIC, Australia; ^16^ Molecular Research and Therapy, Austin Health and University of Melbourne, Heidelberg, VIC, Australia; ^17^ Health and Biosecurity Flagship, The Australian eHealth Research Centre, CSIRO, Victoria, Australia

## Abstract

**Background:**

Centiloid quantification relies on a Standardised Uptake Value Ratio (SUVR) that could be subject to noise, spill‐in, and specific binding in the reference region. We developed a novel deep learning method that learns scan‐specific variability from longitudinal trends to correct SUVR quantification and then validated it in 7 large cohorts.

**Method:**

2,281 participants with 2+ visits in AIBL/ADNI (7,380 scans) had their Amyloid PET images spatially normalised and quantified using the Centiloid SPM8 pipeline. A deep learning network (DeepSUVR) was trained to predict a SUVR correction factor for each spatially normalised image, by penalising unexpected temporal changes based on the deviation from the Centiloid/Year vs mean Centiloid curve. Importantly, while the model requires longitudinal data for training, inference is performed on each visit independently. The model was trained using 5‐fold cross‐validation on ADNI+AIBL and evaluated on the out‐of‐fold AIBL+ADNI as well as OASIS3, AMYPAD, MCSA, HABS‐HD and A4‐LEARN studies (8,806 participants, 12,320 scans), comparing DeepSUVR with standard CL and when using a composite WCb+WM reference region for ^18^F‐Florbetapir.

**Result:**

Fitting a bimodal Gaussian mixture on the baseline CL of each study, DeepSUVR improved the alignment of the first peak across all studies, as well as reducing their standard deviations (Figure 1A) with similar findings across tracers (Figure 1B). DeepSUVR CL had the strongest correlation between baseline CL and MMSE, best group separation between CDR 0/0.5/1 (Table 1A) and highest AUC against visual reads (Table 1B). DeepSUVR improved model fitness (Spearman rank) and inter‐study model agreement in the longitudinal trajectories across studies in both training (Figure 2A) and testing cohorts (Figure 2B). DeepSUVR increased the effect size for the reduced rate of CL increase per year seen with treatment in the A4 study, while reducing the variability in the Amyloid negative subjects (Figure 2C).

**Conclusion:**

Deep learning provides a significant advance in PET quantification of amyloid, outperforming standard methods both cross‐sectionally and longitudinally in both observational and interventional studies. DeepSUVR improves the ability to harmonise large datasets and different PET tracers. This is particularly important for consistent decision making and when the outcome measure of an intervention is expected to be subtle such as altering the rate of accumulation.